# One RNA-binding protein, many decisions: integrating the transcript life cycle into neuronal regulation

**DOI:** 10.3389/fnmol.2025.1716825

**Published:** 2025-11-12

**Authors:** Epaminondas Doxakis, Yuan Chao Xue, Anca F. Savulescu

**Affiliations:** 1 Center of Basic Research, Biomedical Research Foundation of the Academy of Athens, Athens, Greece; 2 Department of Pathology, University of Texas Medical Branch, Galveston, TX, United States; 3 Division of Chemical, Systems and Synthetic Biology, Institute for Infectious Disease and Molecular Medicine, Faculty of Health Sciences, University of Cape Town, Cape Town, South Africa

**Keywords:** RNA-binding proteins, transcript life cycle, alternative polyadenylation, RNA modifications, mRNA localization, local translation, stress

## Introduction

The traditional view that RNA-binding proteins (RBPs) function at single, discrete checkpoints (e.g., splicing, translation) inadequately captures their regulatory complexity, particularly in neurons (Cajigas et al., 2012; Miura et al., 2013; Tushev et al., 2018). A given RBP can act sequentially across the entire transcript life cycle, from transcription and splicing through export, localization, translation, and decay, on long, isoform-diverse transcripts deployed across distinct neuronal compartments. Neuronal phenotypes frequently emerge from coupled regulatory steps rather than isolated checkpoints. A life cycle framework clarifies where an RBP acts on specific neuronal transcripts and how these actions propagate across subsequent regulatory steps.

Synaptic mRNAs carry characteristically long 3′ untranslated regions (3′UTRs) with roughly twice as many predicted miRNA sites per kilobase, expanding the regulatory surface for RBPs and miRNAs (Paschou et al., 2012). During neuronal differentiation, many genes actively lengthen both poly(A) tails and 3′UTRs, highlighting that 3′UTR extension is not a static feature but a regulated neuronal program (Miura et al., 2013; Kiltschewskij et al., 2023). This increased regulatory complexity necessitates systematic approaches that track RBP actions across checkpoints rather than attributing effects to single regulatory steps. Adopting a life cycle perspective prevents misattribution by identifying where along the regulatory pathway an RBP influences specific neuronal transcripts.

### Scope and use of non-neuronal data

Although most foundational studies of RBP mechanisms have been conducted in non-neuronal systems, the core regulatory principles are largely conserved across cell types (Corley et al., 2020; Engel et al., 2020). Where neuronal-specific data exist, these are prioritized; non-neuronal findings are included when they illuminate fundamental mechanisms likely operative in neurons.

### Mechanistic checkpoints across the transcript life cycle

#### Regulatory commitment begins in the nucleus

Several RBPs function at transcriptional checkpoints before any post-transcriptional decisions occur. For instance, heterogeneous nuclear ribonucleoprotein A1 (hnRNPA1) binds single-stranded DNA and G-quadruplex-forming promoter regions and engages the 7SK-HEXIM-P-TEFb axis to influence RNA polymerase II (RNAPII) pause-release and elongation (Zhang et al., 2006; Barrandon et al., 2007; Nishikawa et al., 2019). RNAPII elongation rate directly influences exon choice and 3′-end selection. Reduced elongation shifts poly(A) site usage toward proximal sites, whereas accelerated elongation favors distal sites *in vivo* (Geisberg et al., 2020; Yague-Sanz et al., 2020). In parallel, U1 small nuclear ribonucleoprotein (U1 snRNP)-mediated “telescripting” suppresses premature cleavage and polyadenylation across nascent RNAs, including long introns, thereby preserving full-length pre-mRNA and shaping alternative polyadenylation (APA) outcomes (Kaida et al., 2010; Berg et al., 2012; So et al., 2019).

Nuclear factors subsequently bias 3′-end choice in neurons. Cleavage factor Im complex 25 kDa subunit (CFIm25, NUDT21) promotes distal polyadenylation; reduced CFIm25 levels shift usage toward proximal sites, shorten 3′UTRs in the mouse hippocampus, and produce learning deficits alongside cortical hyperexcitability *in vivo* (Alcott et al., 2020). In Drosophila, embryonic lethal abnormal visual-like (ELAV/Hu) paralogs drive global neuronal 3′UTR extension (Hilgers et al., 2012; Oktaba et al., 2015). In mammals, neuronal ELAVLs (ELAV2-4, HuB/C/D) regulate alternative polyadenylation at defined loci such as *ELAVL1 (HuR)*, where distal site usage during differentiation produces long 3′UTR isoforms with reduced translation and stability (Dai et al., 2012; Mansfield and Keene, 2012). Recent work identifies PQBP1 as a regulator of APA in neural progenitor cells. PQBP1 interacts with UGUA motifs and can impede recruitment of the CFIm complex, maintaining cell-specific poly(A) site profiles and balancing progenitor proliferation and differentiation (Liu et al., 2024).

These 3′UTR decisions have direct functional consequences. At the brain-derived neurotrophic factor (*Bdnf*) locus, short- and long-3′UTR isoforms follow different routes: the short isoform remains in the soma, whereas the long isoform localizes to dendrites and supports local functions in hippocampal neurons (An et al., 2008). The long 3′UTR imposes translational restraint at baseline but permits rapid activity-dependent translation, in contrast to the constitutively active short 3′UTR (Lau et al., 2010). Neurotrophins and distinct RBP assemblies further modulate dendritic targeting of these *Bdnf* isoforms (Vicario et al., 2015).

#### Export is not a neutral handoff: nuclear assembly decisions carry through to shape cytoplasmic fate

The transcription and export (TREX) complex assembles during splicing and 3′-end processing, licensing nuclear RNA export factor 1 (NXF1/TAP) to bind mature messenger ribonucleoproteins (mRNPs) and thereby coupling nuclear processing to export competence and downstream cytoplasmic availability (Viphakone et al., 2012; Puhringer et al., 2020). Co-transcriptional deposition of the exon junction complex at exon-exon junctions provides a platform that interfaces with NXF1 loading, linking splicing to export and downstream surveillance mechanisms such as nonsense-mediated decay and translation enhancement (Le Hir et al., 2000; Viphakone et al., 2019).

Export routes can switch under defined cellular stresses. During heat shock, HuR -mediated RNA export shifts to a chromosome region maintenance 1/exportin 1 (CRM1/XPO1)-dependent route via its shuttling ligands pp32/ANP32A and APRIL/ANP32B; under these conditions export becomes leptomycin B-sensitive, and CRM1 co-immunoprecipitates with HuR only following heat shock (Gallouzi et al., 2001). In addition, after nuclear export, 3′UTRs can undergo remodeling through endonucleolytic cleavage. For instance, cytoplasmic cleavage of the inositol monophosphatase 1 (*IMPA1*) 3′UTR generates a more translatable isoform, which is required for maintaining axon integrity (Andreassi et al., 2021).

#### Localization and translation are often co-regulated in neurons

Many neurite-bound transcripts carry RNA G-quadruplex motifs that recruit fragile X mental retardation protein (FMRP). FMRP promotes projection-side localization while stalling elongation, coupling transport to translational restraint with stimulus-triggered release (Darnell et al., 2011; Goering et al., 2020). Among cytoskeletal transcripts subject to FMRP-linked ribosome stalling in neurons is *MAP1B* (Darnell et al., 2011). Further, local translation and mitochondrial tethering of *Pink1* mRNA by synaptojanin 2 binding protein (SYNJ2BP) and synaptojanin 2 (SYNJ2A) are required for activation of the PINK1/Parkin pathway in axons (Harbauer et al., 2022).

Localization codes operate within neuronal transcripts. The β*-actin* zipcode bound by zipcode-binding protein 1 (ZBP1/IGF2BP1) mediates dendritic transport and local translation, and the A2 response element recognized by hnRNPA2/B1 supports activity-regulated dendritic delivery (Eom et al., 2003; Shan et al., 2003; Patel et al., 2012; Leal et al., 2014). Coding regions can also direct targeting. In motor neuron axons, *Cox7c* mRNA co-transports with mitochondria through a mechanism that depends on the coding region, not the 3′UTR (Cohen et al., 2022). Mechanistically, RBPs couple cargo to motors via adaptors such as adenomatous polyposis coli (APC), which links select mRNAs to kinesin complexes for bidirectional transport in neurons (Baumann et al., 2022).

This cross-regulatory coordination appears in neuron-focused crosslinking and immunoprecipitation (CLIP) datasets, which show RBP occupancy spanning splicing enhancers and 3′UTRs, indicating interactions across multiple regulatory steps. In the brain, the RBP neuro-oncological ventral antigen (NOVA) binds splice-regulatory elements and 3′UTRs, primarily regulating neuronal splicing programs while also affecting alternative polyadenylation at select loci (Licatalosi et al., 2008). RNA-binding FOX protein (RBFOX) family binding maps define intronic sites that predict neuronal splicing programs; additionally, cytoplasmic RBFOX1 binds 3′UTRs in neurons and increases target mRNA stability (Weyn-Vanhentenryck et al., 2014; Lee et al., 2016).

Noncoding RNAs add regulatory complexity. Long non-coding RNA (lncRNA)-mRNA base pairing through inverted Alu elements can create Staufen-binding sites in trans, triggering Staufen-mediated decay (SMD) (Gong and Maquat, 2011). Moreover, circular RNAs (circRNAs) can escort RBPs to their targets, as demonstrated by circNSUN2 forming a ternary complex with IGF2BP2 and *HMGA2* mRNA that stabilizes the message (Chen et al., 2019).

#### RNA modifications further modulate these relationships

In the hippocampus, m6A reader YTH N6-methyladenosine RBP F1 (YTHDF1) enhances translation of synaptic transcripts in an activity-dependent manner and is required for learning and long-term potentiation (Shi et al., 2018). In the nucleus, the m6A modification increases HNRNPC access by locally relaxing RNA structure, which alters splicing and RNA abundance, linking epitranscriptomic marks to RBP occupancy and downstream processing (Liu et al., 2015). Additional modifications provide export- and binding-sensitive regulatory levers. m5C deposition by NOP2/Sun RNA methyltransferase (NSUN2) and recognition by Aly/REF export factor (ALYREF) promote mRNA export (Yang et al., 2017). mRNA pseudouridylation represents another widespread modification that increases under serum starvation; TruB pseudouridine synthase family member 1 (TRUB1) serves as a major contributor to mRNA pseudouridine in mammalian cells (Carlile et al., 2014; Safra et al., 2017). When pseudouridine occurs at stop codons, it can reduce termination efficiency (Karijolich and Yu, 2011). These examples illustrate how chemical modifications and cellular conditions can reroute the same transcript through different regulatory outcomes. The modifications create context-dependent switches that alter RBP binding patterns and downstream transcript processing, demonstrating another layer of life cycle regulation beyond protein-RNA interactions alone.

#### A single-gene paradigm illustrates how regulatory steps combine

AU-rich element RNA binding protein 1 (AUF1/HNRNPD) binds proximal and distal elements in the *SNCA* 3′UTR and coordinates multiple regulatory steps in cellular systems: it influences pre-mRNA maturation, is necessary for efficient nuclear export, promotes deadenylation-linked decay of isoforms with shorter 3′UTRs, and reduces ribosome engagement, together lowering α-synuclein output (Kattan et al., 2023). Within this same transcript, Pumilio RNA-binding protein (PUM1) binds two conserved sites in the *SNCA* 3′UTR and preferentially suppresses the long 3′UTR isoform. PUM1 redistributes *SNCA* between soma and axons, normalizes α-synuclein levels in patient-derived neurons with *SNCA* locus triplication, and modulates microRNA responsiveness, indicating coordinated control across isoform choice, subcellular routing, and post-transcriptional sensitivity (Cabaj et al., 2025). This single-locus analysis demonstrates how multiple RBPs can coordinate transcript processing, export, stability, localization, and translation to control protein output through interconnected rather than independent mechanisms.

#### Stress conditions rewire RBP-mRNA partnerships and translation

The RBPs T-cell intracellular antigen 1 (TIA1) and TIA1-related protein TIAR (TIAL1) accumulate in the cytoplasm during mild heat shock and nucleate stress granules (SGs) when eukaryotic initiation factor 2 alpha (eIF2α) becomes phosphorylated (Kedersha et al., 1999). Additionally, TIA1 oxidation decreases SG assembly and increases sensitivity to apoptosis (Arimoto-Matsuzaki et al., 2016). The TIA1 proximity interactome reveals that TIA1 partners shift between basal conditions and sodium-arsenite stress, indicating that the protein machinery associated with specific RNAs adapts to cellular conditions (Gourdomichali et al., 2022). During osmotic shock, hnRNPA1 becomes hyperphosphorylated and accumulates in the cytoplasm (Allemand et al., 2005). Under sodium arsenite, heat shock, or hyperosmotic stress, hnRNPA1 also relocates to SGs (Guil et al., 2006). Depletion of nuclear hnRNPA1 alters alternative splicing, linking stress-driven relocalization of splicing regulators to splicing control (Allemand et al., 2005). Phase-separated condensates formed by RBPs represent distinct regulatory compartments that reorganize under stress (Brangwynne et al., 2009; Lin et al., 2024).

At the level of output, translation during stress becomes selectively altered for defined transcript subsets. When eIF2α is phosphorylated, mRNAs with upstream open reading frames (uORFs) in their 5′ leaders, such as activating transcription factor 4 (*ATF4*), undergo preferential translation via delayed reinitiation (Harding et al., 2000; Vattem and Wek, 2004). When cap-dependent initiation becomes attenuated, cap-independent translation via internal ribosome entry site (IRES)-like elements can sustain protein output; the *SNCA* 5′UTR supports such activity (Koukouraki and Doxakis, 2016). Through a distinct pathway, mechanistic target of rapamycin complex 1 (mTORC1) inhibition triggers La-related protein 1 (LARP1)-dependent repression of 5′-terminal oligopyrimidine (5′-TOP) mRNAs (Fonseca et al., 2015; Philippe et al., 2020).

These stress-responsive mechanisms illustrate how cellular conditions can rapidly reconfigure RBP-transcript relationships and redirect the same transcripts through alternative regulatory pathways, thereby introducing temporal dynamics to the life cycle framework.

#### An experimental design that captures cross-regulatory coordination

**Figure 1:**
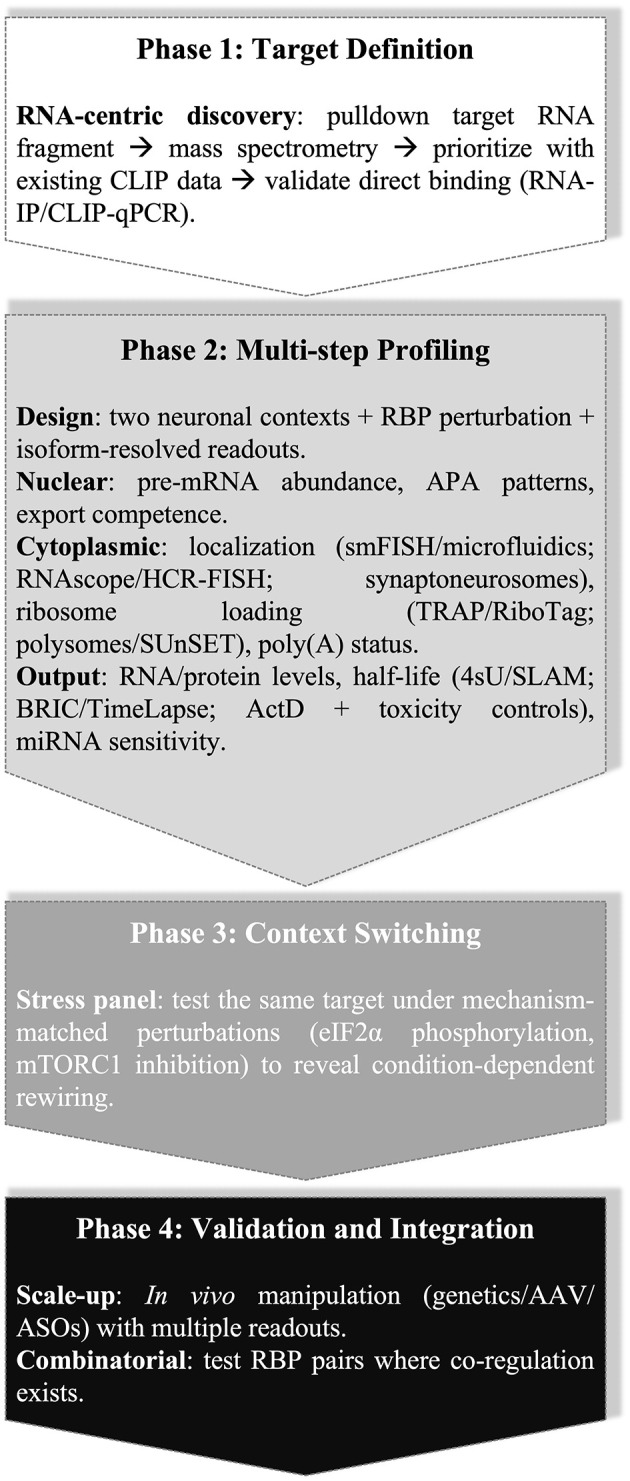
Four-phase, isoform-aware workflow for mapping RBP control across the transcript life cycle in neurons.

A practical framework that tracks mRNA transcripts across regulatory steps (transcription, processing, export, localization, translation, decay) captures complex regulation more accurately than relying on single assays (Figure 1). Integrating information from different experimental approaches is essential to understanding the broader regulatory scheme controlled by RBPs in neurons. The experimental design outlined below can be repeated under defined stress conditions when appropriate, to separate primary from secondary effects and assign mechanisms to the correct regulatory step of the transcript life cycle (Figure 1).

Begin with RNA-centric candidate discovery for a defined target transcript. Perform biochemical pulldown of a 3′UTR segment or exon, followed by mass spectrometry. Prioritize candidates using CLIP-class binding evidence where available, and confirm direct binding on endogenous RNA by RNA immunoprecipitation (RNA-IP) or CLIP-qPCR (Yoon and Gorospe, 2016; Kattan et al., 2023). Proceed to an isoform-aware experimental panel using two neuronal contexts that differ in baseline RBP expression or RNA metabolism. Following relevant RBP perturbation, quantify pre-mRNA (transcriptional input), mature isoforms (splicing and alternative polyadenylation outcomes), and nuclear-cytoplasmic distribution with fraction-purity controls (e.g., *MALAT1* and/or *RNU6* for nuclear; *GAPDH* mRNA for cytoplasm), using conventional fractionation with isoform-specific RT-qPCR or RNA-seq, and assess subcellular localization using single-molecule FISH (smFISH) (Raj et al., 2008), axon-soma microfluidics (Taylor et al., 2005), or synapse-targeted micro-local perfusion (microLP) (Taylor et al., 2010). When smFISH or microfluidics are unavailable, synaptoneurosome preparations provide synapse-enriched RNA for biochemical assays (Westmark et al., 2011). RNAscope (Wang et al., 2012) and hybridization chain reaction (HCR) FISH (Choi et al., 2014) are additional *in situ* options.

Profile ribosome engagement using FLAG-RPL22 IP (Kattan et al., 2023), translating ribosome affinity purification (TRAP) (EGFP-L10a) (Heiman et al., 2014), or ribosomal tagging (RiboTag) (HA-RPL22) in Cre-defined neurons (Sanz et al., 2009), followed by quantification of isoforms using distal 3′UTR primers. Classical sucrose-gradient polysome profiling with isoform-specific qPCR is an effective substitute (Chasse et al., 2017), and SUnSET provides a complementary non-genetic readout of protein synthesis (Schmidt et al., 2009).

To integrate transcript stability with translation effects, measure poly(A) tail lengths using the extended poly(A) test (ePAT), and analyze isoform-specific tail-length shifts alongside ribosome engagement, half-life, and protein output (Janicke et al., 2012; Subtelny et al., 2014; Lima et al., 2017). Test miRNA sensitivity, since 3′UTR switching can expose or occlude binding sites, and RBPs can remodel Ago proteins access through competition or structural changes (Bhattacharyya et al., 2006; Surgucheva et al., 2013; Kim et al., 2021; Cabaj et al., 2025).

For decay measurements, avoid treatments that disrupt endogenous recognition and execution steps. In mammalian cells, PAN2-PAN3 initiates poly(A) shortening while CCR4-NOT cooperates with poly(A)-binding protein to complete deadenylation (Uchida et al., 2004; Yi et al., 2018). Shortened tails permit decapping by DCP2 assembled on the EDC4 scaffold with DCP1, and decapped RNA undergoes 5′ to 3′ degradation by 5′-3′ exoribonuclease 1 (XRN1) (Chang et al., 2014; Brothers et al., 2023). Estimate isoform-specific half-lives using 4sU pulse-chase coupled to thiol(SH)-linked alkylation for the metabolic sequencing of RNA (SLAM-seq) or SLAM-qPCR, which preserves translation during measurement (Herzog et al., 2017). Alternative approaches include bromouridine immunoprecipitation chase sequencing (BRIC-seq) or BRIC-qPCR with 5-bromouridine labeling (Tani et al., 2012) and TimeLapse-seq for chemical recoding of 4sU (Schofield et al., 2018). Where chemistry or platforms are constrained, conventional actinomycin D chase with qPCR provides decay estimates, but interpretation requires toxicity controls such rRNA processing markers and p53-responsive transcripts (Bensaude, 2011). Avoid global translation inhibitors (e.g., cycloheximide) when assaying translation-dependent decay, since they suppress SMD and nonsense-mediated decay (NMD); instead, infer decay from labeling methods or brief, toxicity-controlled transcriptional blocks (Gong and Maquat, 2011).

When stress-dependence appears likely, repeat the isoform-aware panel under defined, mechanism-matched perturbations: heat shock to probe CRM1-dependent nuclear export switches (Gallouzi et al., 2001); sodium arsenite for eIF2α phosphorylation and stress-granule formation (Kedersha et al., 1999; Gourdomichali et al., 2022); mTORC1 inhibition to test 5′-TOP control via LARP1 (Fonseca et al., 2015; Philippe et al., 2020); cap-dependent attenuation to assay IRES-mediated translation, such as the *SNCA* 5′UTR (Koukouraki and Doxakis, 2016); or ER stress (e.g., thapsigargin) to test uORF-dependent translation (ATF4) (Harding et al., 2000; Vattem and Wek, 2004).

Two extensions add mechanistic weight once single-factor effects are mapped. First, test *in vivo* by altering RBP levels with conditional genetics, focal adenoviral-associated viruses (AAV) delivery, or intracerebroventricular antisense oligonucleotides, and measure the same pre-mRNA, isoform, localization, ribosome engagement, and protein endpoints in targeted brain regions or cell types. Second, when two RBPs independently regulate the same target, consider pairwise perturbations guided by co-occupancy evidence and mechanistic precedent, as shown for neuronal ELAVLs and AUF1 on *APP* (Fragkouli et al., 2017).

## Future technological integration

Single-nucleus RNA-seq and spatial transcriptomics add cell-type and spatial context to the isoform-aware panel, enabling tissue-level maps of neuronal programs and RNA localization, and placing RBP effects in their native cellular niches (Chen et al., 2015; Hu et al., 2017; Rodriques et al., 2019; Booeshaghi et al., 2021). Patient-derived iPSC neurons with mutant transactive response DNA binding protein 43 kDa (TDP-43) or fused in sarcoma (FUS) show disease-relevant phenotypes (Bilican et al., 2012; Devlin et al., 2015; Higelin et al., 2016). In these human neurons, genome-scale CRISPRi screens can identify modifier genes and compensatory pathways (Tian et al., 2019). Long-read/native RNA sequencing resolves full-length isoforms on single molecules and can report RNA modifications, exposing RBP-sensitive isoform choices and marks that alter RBP binding (Garalde et al., 2018; Workman et al., 2019; Begik et al., 2021).

## Concluding remarks

Neuronal RNA regulation operates through coupled, sequential steps where early processing choices constrain later outcomes and where effects at one checkpoint propagate through subsequent stages.

Early decisions made in the nucleus set the stage for what follows. U1-guided protection and 3′-end choice shape isoforms; TREX and the exon junction complex link splicing and 3′-end formation to export competence; export routes can switch during stress; and post-export remodeling, including 3′UTR cleavage, can change local availability. Together, these events carry forward to influence cytoplasmic fate.

In the cytoplasm, localization and translation are often coupled. Transport factors can hold ribosomes in check during transit and release them with activity. Decay frequently depends on translation status. The *SNCA* example shows how more than one RBP can coordinate processing, export, stability, localization, ribosome engagement, and protein output at a single locus. Chemical modifications and noncoding RNAs add context-dependent layers that tune these relationships.

Two considerations are central. CLIP occupancy alone does not prove function, and effects are commonly isoform-specific and cell-type-specific. The practical answer is to pair defined perturbations with isoform-aware, subcellularly resolved, ribosome-informed readouts in at least two neuronal backgrounds, and to repeat the panel under relevant stress conditions. This approach moves work from isolated events to integrated lifecycle control, encouraging experiments that follow the same RNA across regulatory stages within defined cellular contexts.
